# When you avoid your feelings, you may feel even worse: how depersonalization puts you at risk of depression

**DOI:** 10.3389/fpsyt.2024.1481439

**Published:** 2024-10-17

**Authors:** Dominika Fortuna, Krystyna Golonka

**Affiliations:** ^1^ Doctoral School in the Social Sciences, Jagiellonian University, Krakow, Poland; ^2^ Department of Crisis Intervention and Psychotherapy, Institute of Applied Psychology, Faculty of Management and Social Communication, Jagiellonian University, Krakow, Poland

**Keywords:** depersonalization, depression, emotions, regulation strategies, non-clinical sample, measurement of depersonalization

## Abstract

**Background:**

The clinical form of depersonalization affects approximately 1%–2% of the adult population. This study aimed to describe the symptoms of depersonalization in a non-clinical sample and to operationalize depersonalization as a regulatory mechanism. This article introduces the Depersonalization Mechanism Scale, 41-item measure developed to assess one’s tendency for depersonalization in response to overstimulation. The aim of the study is to explore how depersonalization mechanism is associated with cognitive and behavioral emotion regulation strategies, depression, and anxiety.

**Method:**

The study included a sample of 300 Polish adults (149 men) from the general population, ranging in age from 18 to 60. Participants were administered the following questionnaires: Depersonalization Mechanism Scale (DMS), Behavioral Emotion Regulation Questionnaire (BERQ), Cognitive Emotion Regulation Questionnaire (CERQ), Occupational Depression Inventory (ODI), Patient Health Questionnaire (PHQ), and Trait Anxiety Scale (SL-C).

**Results:**

An exploratory factor analysis revealed a two-factor structure of Depersonalization Mechanism Scale, with very high reliability coefficients for both subscales and full scale. A regression analysis revealed that depersonalization mechanism is a significant predictor of depressive symptoms. Depersonalization mechanism is strongly correlated with maladaptive regulation strategies such as withdrawal, ignoring, rumination, catastrophizing, self-blame, and blaming others. Weaker but significant connections were identified with certain adaptive strategies: acceptance, positive refocusing, putting into perspective, and seeking social support. Women were more prone to depersonalization than men.

**Conclusions:**

Further research on depersonalization in non-clinical samples may improve understanding of this mechanism in the general population. This knowledge, combined with greater education about non-clinical forms of depersonalization, may support preventive programs against depression and professional assistance for people facing acute or chronic stressful life events.

## Introduction

Difficulties in emotion regulation play an important role in the development and maintenance of psychopathology. Emotional dysregulation can be found in many mental health problems, including substance abuse, eating disorders, depression, or borderline personality disorder (1–4). Various definitions of emotion regulation can be found across literature, one of the most influential being the one proposed by Gross (5, 6). It is broadly defined as processes through which individuals monitor, evaluate, and modulate their emotions to adequately respond to environmental demands. Utilizing this framework, Folkman and Lazarus (7) introduced a distinction between problem- and emotion-focused coping, emphasizing the more adaptive value of problem-solving strategies. Gross’s model is especially useful in investigating the relationship between specific strategies and symptoms of clinical disorders (1, 4, 8, 9). Maladaptive strategies (e.g., rumination, suppression, or avoidance) are consistently found to be more strongly associated with anxiety, depression, eating disorders, and substance abuse (10, 11). To specify more precisely the subtleties among strategies, Garnefski et al. (12) and Kraij and Garnefski (13) proposed a distinction between cognitive and behavioral strategies. While catastrophizing, rumination, self-blame, and other-blame are seen as less adaptive, positive reappraisal, planning, and putting into perspective are described as more adaptive (14) and useful in dealing with life difficulties, depression, anxiety, or anger (15). Behavioral strategies also can be divided in the same way—adaptive strategies are seeking distraction, actively approaching, and seeking social support, as opposite to less adaptive withdrawal and ignoring. While some strategies are well described and studied, some remain unclear in terms of definition, functions, and adaptive value. Another problem is associated with grouping strategies in complex mechanisms such as depersonalization. We propose a different understanding of this phenomenon and investigate its relationship to specific emotion regulation strategies, anxiety, and depression.

In its clinical form, depersonalization is characterized by persistent or recurrent episodes of experiences of unreality, detachment, or being an outside observer of one’s thoughts, feelings, sensations, body, or actions (16). Two core components of depersonalization are detachment and hypoemotionality (emotional numbing or blunting) (17). An individual may feel as if in a dream or a game and may have a feeling of alienation from the reflection in the mirror. On a conscious level, a person recognizes themselves (“I know it is me”) but lacks an emotional connection to their image (“It doesn’t feel like me”), resulting in a profound sense of strangeness. Hypoemotionality refers to blunted affect: an individual remains capable of expressing emotions yet experiences them as strangely dampened.

This phenomenon can be described on a continuum, ranging from chronic, clinical form (depersonalization/derealization disorder), to transient episodes. Short-term experiences of depersonalization are mostly triggered by fatigue, anxiety, substance abuse, stress (18), or artificial induction (19) and are considered universal in the general population (20). Depersonalization appears in order to preserve adaptive behaviors (21) and allows to put off emotions and feelings that are too difficult to cope with and, therefore, tolerate the circumstances one is in (22).

We conceptualize depersonalization as an emotion regulation mechanism, broader than specific strategies. The use of word mechanism instead of strategy is not accidental—we suggest that a person can have at least some amount of control over which strategy to use in certain situation, while depersonalization is independent of the will. In the face of an overwhelming or demanding situation, depersonalization can “switch on” and provide a temporary relief. It distances an individual from their emotions and enables them to tolerate the challenges as long as needed. Despite short-term relief, it can exacerbate distress in the long run, potentially leading to emotional depletion, difficulties in maintaining relationships, and overall psychological distress. Regardless from clinical domain, we took inspiration from the burnout syndrome, where depersonalization is one of three core dimensions (23). In burnout, it is characterized as an increased mental distance to work, negativity, and work-related cynicism (24). On the one hand, clinical depersonalization withdraws a person from themselves; on the other, in burnout concept, it withdraws a person from other people and work-related context (25). We decided to merge these two ways of thinking and propose that trait-like depersonalization dampens emotional experiences and interferes with the ability to maintain personal relationships. If an individual has a problem accessing their emotions, feels distanced, it is hard to be empathetic and attentive. As a result, they have very little energy to engage in relationships and provide support or reciprocation of any kind. We believe that this type of depersonalization could be placed in the middle of the abovementioned continuum, offering a more comprehensive understanding of this phenomenon. We conceptualize it as a trait-like tendency to “activate” depersonalization in order to regulate one’s emotional state, which means certain individuals may be more prone to it than the others.

Since existing questionnaires focus on a clinical form of depersonalization, we decided to develop a new measurement tool (see *Materials and methods*). The aim of this paper is fourfold: (1) to propose a novel understanding of depersonalization as an emotion regulation mechanism; (2) to investigate relationships between depersonalization mechanism, cognitive and behavioral emotion regulation strategies, depression symptoms, and trait anxiety; (3) to explore behavioral and cognitive emotion regulation strategies as predictors of depersonalization mechanism; and 4) investigate whether depersonalization mechanism is a predictive factor for depression symptoms.

## Materials and methods

### Participants

The sample consisted of 300 Polish participants from the general population, meeting inclusion criteria: age 18–60. All participants were recruited by the Polish national research panel. Sample characteristics according to gender, age, and education are as follows: 151 women (50.3%) and 149 men (49.7%); 102 persons (34%) were between 46 and 60 years old, 86 persons (28.7%) between 36 and 45 years old, 70 persons (23.3%) were between 26 and 35 years old, and 42 persons (14%) were between 18 and 25 years old; 129 participants (43%) have received higher education, 167 persons (55.7%) finished high school or similar type of education, and four participants (1.3%) finished primary education. A total of 226 people (75.3%) were professionally active. Participants were asked to disclose any health problems; 151 participants (50.3%) had chronic illnesses (predominantly diabetes, high blood pressure, and asthma), and 146 participants (48.7%) were taking medication (mostly related to chronic illnesses). The sample was representative across all provinces of Poland.

### Design and procedure

The research was carried out by the national research panel. At the beginning, respondents were asked about their gender, age, education, chronic illnesses, currently taken medication, and family risk factors. Then, they were provided with a set of questionnaires, described in detail in *Measures*. This study was carried out in accordance with the recommendations of the APA Ethics Code and Helsinki Declaration.

We constructed a new questionnaire that measures a tendency for depersonalization, named Depersonalization Mechanism Scale (DMS). During the developmental phase, we performed a pilot study, starting with an initial pool of 76 items. Items were inspired by already existing depersonalization scales, presenting satisfactory psychometric values: Dissociative Experiences Scale (26), Cambridge Depersonalization Scale (27), Depersonalization–Derealization Inventory (28), The Perceptual Alteration Scale (29), and The Dissociation Questionnaire (30). Moreover, some of the items were inspired by depersonalization subscales from burnout questionnaires: The Maslach Burnout Inventory–General Survey (MBI-GS) (23) (Cynicism subscale), Oldenburg Burnout Inventory (Disengagement subscale), and Link Burnout Questionnaire (25) (Deterioration of relations subscale). Initial 76 items were divided into three categories: 1) selected from the existing scales, for example, “I find my mind blank” from The Perceptual Alteration Scale (29); 2) items selected from the existing scales but with altered phrasing, for example, “I have the experience (…) instead of “Some people have the experience (…)” from Dissociative Experiences Scale (26); 3) created by the authors for example, “I feel that I have so little mental energy that I am able to do bare minimum when it comes to interacting with other people.” After the pilot study, redundant and weak loading items were reviewed or discarded. The 50-item version remained and was utilized during research. This version has undergone another psychometric evaluation and factor analysis (described in detail below, in *Results*, *Factor analysis*). The final version of the scale consists of 41 items (described in detail in the *Measures*). The scale is designed as self-report and can be administered to adults. A whole version of the scale is presented in **Supplementary Materials**.

As the aim of this study was to explore the relationships between depersonalization mechanism, cognitive and behavioral emotion regulation strategies, depression symptoms, and trait anxiety, several instruments were used: Cognitive Emotion Regulation Questionnaire and Behavioral Emotion Regulation Questionnaire to analyze associations with different emotion regulation strategies; Patient Health Questionnaire and Occupational Depression Inventory for employees to assess links with depression; and Trait Anxiety Scale to investigate the relationship between depersonalization mechanism and anxiety.

### Measures

#### Depersonalization Mechanism Scale (DMS)

This is a 41-item, self-report measure designed to assess a tendency to depersonalization. It consists of two subscales: emotional numbness and detachment. Items are rated on 5-point Likert scale, ranging from 0 never to 4 always. A general score ranges from 0 to 164 with a higher score, indicating a higher tendency to depersonalization. The scale was originally constructed in Polish and translated into English by the authors. Reliability scores are presented in **Table 1**.

**Table 1:** Reliability scores for the Depersonalization Mechanism Scale (DMS).

	Detachment	Emotional Numbness	Depersonalization
Cronbach’s *α*	0.94	0.97	0.97
Average variance extracted (AVE)	0.46	0.44	0.45
McDonald’s omega	0.97	0.94	0.97
Guttman’s lambda-4	0.94	0.91	
Spearman–Brown correction	0.94	0.91	

#### Cognitive Emotion Regulation Questionnaire

Cognitive Emotion Regulation Questionnaire (CERQ) (31) is a 36-item, self-report measure designed to assess nine cognitive emotion regulation strategies used in response to threatening or stressful life events. It consists of nine, four-item scales: self-blame, blaming others, acceptance, refocusing on planning, positive refocusing, rumination, positive reappraisal, putting into perspective, and catastrophizing. Items are rated on a 5-point Likert scale, ranging from 1 (almost) never to 5 (almost) always. Subscale scores range from 4 to 20 with higher scores indicating greater tendency to particular strategy. The psychometric characteristics of the original version indicate good reliability, with Cronbach’s *α* ranging from 0.75 to 0.86. In this study, Cronbach’s *α* of the Polish version of CERQ ranges from 0.73 to 0.85.

#### Behavioral Emotion Regulation Questionnaire

Behavioral Emotion Regulation Questionnaire (BERQ) (13) is a 20-item, self-report measure to designed to describe five behavioral coping strategies: seeking distraction, withdrawal, actively approaching, seeking social support, and ignoring. Each scale consists of four items rated on 5-point scale, ranging from 1 (almost) never to 5 (almost) always. Each subscale is scored from 4 to 20—the higher the scores, the stronger the behavioral strategy. The psychometric characteristics of the original version indicate good reliability, with Cronbach *α* ranging from 0.86 to 0.93 (13). In this study, Cronbach’s *α* of the Polish version of BERQ ranges from 0.80 to 0.94.

#### Occupational Depression Inventory

Occupational Depression Inventory (ODI) (32) is a nine-item, self-report measure designed to assess the severity of work-attributed depressive symptoms. It focuses on nine areas of depressive episodes (consistent with DSM-5 diagnostic criteria for major depressive disorder): anhedonia, depressed mood, sleep alterations, fatigue/loss of energy, appetite alterations, feelings of worthlessness, cognitive impairment, psychomotor alterations, and suicidal ideation. Additional question relates to work-related cause of depressive symptoms. Items are rated on a 4-point scale, ranging from 0 never or almost never to 3 nearly every day. In this study, Cronbach’s *α* of the Polish version is 0.94.

#### Patient Health Questionnaire

Patient Health Questionnaire (PHQ-9) (33) is a nine-item self-report measure to assess the depressive symptoms. Participants are asked how often they experienced described states during the last 2 weeks. Items are rated on a 4-point scale, ranging from not at all to nearly every day, and are scored from 0 to 3, respectively. The general scores range from 0 to 27 and refer to different levels of depression severity (from minimal to severe). In previous studies, Cronbach’s *α* revealed good reliability (e.g., 0.89) (34). In this study, Cronbach’s *α* is 0.92.

#### Trait Anxiety Scale—SL-C

Trait Anxiety Scale—SL-C (35) is a 15-item measure assessing the intensity of anxiety as a personality trait. This is an English version of a Polish scale (Skala Lęku–Cecha, SL-C). Trait anxiety is understood as a tendency to perceive a situation as threatening or to anticipate future events in terms of the danger that manifests through characteristic cognitive, emotional, and behavioral symptoms. Items are rated on a 4-point scale, from 3 (often) to 0 (never). The SL-C is a one-factor tool; the score ranges from 0 (minimum trait anxiety intensity) to 45 (maximum trait anxiety intensity). The original Cronbach’s *α* coefficient was 0.86. In this study, Cronbach’s *α* is 0.89.

## Results

The analysis was carried out with IBM SPSS Statistics 25. The following analysis was performed: descriptive statistics with Kolmogorov–Smirnov test, Pearson *r* correlations, Student’s *t*-test for independent samples, Kruskal–Wallis test, and multiple regression analysis. Significance level was set at *α* = 0.05.

### Descriptive statistics

In the first step, descriptive statistics were calculated, along with Kołmogorov–Smirnov tests to determine distribution of variables. Apart from the emotional numbness subscale, all subscales significantly differed from normal distribution. Additional skewness tests were performed in order to verify whether they are between −/+2 standard deviations from the mean. In that case, it is safe to assume that the distribution is not significantly different from normal distribution (36). Based on the results presented in **Table 2**, it was decided to use parametric tests while fulfilling other assumptions.

**Table 2:** Descriptive statistics of the used methods.

Method	Subscale	M	Me	SD	Sk.	Kurt.	Min.	Max	W	*p*
DMS	Detachment	1.28	1.19	0.82	0.40	−0.63	0	3.52	0.07	<0.001
	Emotional numbness	1.78	1.79	0.82	0.15	−0.27	0	3.86	0.04	0.200
	Depersonalization	1.45	1.41	0.78	0.30	−0.49	0	3.54	0.06	0.011
BERQ	Seeking distraction	2.69	2.75	0.80	0.44	0.36	1	5	0.10	<0.001
	Withdrawal	2.30	2	1.11	0.86	−0.12	1	5	0.18	<0.001
	Actively approaching	2.79	2.75	1	0.48	−0.37	1	5	0.10	<0.001
	Seeking social support	2.45	2.25	0.95	0.75	0.21	1	5	0.13	<0.001
	Ignoring	2.31	2	0.92	0.75	0.27	1	5	0.13	<0.001
CERQ	Self-blame	2.56	2.50	0.79	0.71	0.30	1	5	0.14	<0.001
	Acceptance	2.66	2.50	0.78	0.63	0.53	1	5	0.14	<0.001
	Rumination	2.67	2.50	0.80	0.40	−0.12	1	5	0.10	<0.001
	Positive refocusing	2.52	2.50	0.80	0.63	0.35	1	5	0.12	<0.001
	Refocus on planning	2.91	2.75	0.92	0.46	−0.32	1	5	0.11	<0.001
	Positive reappraisal	2.63	2.50	0.90	0.39	−0.08	1	5	0.10	<0.001
	Putting into perspective	2.70	2.50	0.89	0.53	−0.02	1	5	0.13	<0.001
	Catastrophizing	2.41	2.25	0.86	0.78	0.71	1	5	0.13	<0.001
	Blaming others	2.23	2	0.82	0.97	1.39	1	5	0.15	<0.001
ODI	Occupational depression	0.71	0.44	0.76	1.07	0.34	0	3	0.18	<0.001
PHQ	Depression (PHQ)	0.89	0.67	0.76	0.72	−0.37	0	3	0.14	<0.001
	Difficulty with work, housework or relationships with other people	1.81	2	0.75	0.71	0.25	1	4	0.25	<0.001
SL-C	Anxiety-a trait	39.21	40	8.62	−0.21	−0.53	17	58	0.06	0.005

M, mean; Me, median; SD, standard deviation; Sk., skewness.; Kurt., kurtosis; Min and Max., minimum and maximum value; W, Kołmogorov–Smirnov test; p, significance level; DMS, Depersonalization Mechanism Scale; BERQ, Behavioral Emotion Regulation Questionnaire; CERQ, Cognitive Emotion Regulation Questionnaire; ODI, Occupational Depression Inventory; PHQ, Patient Health Questionnaire; SL-C, Trait Anxiety Scale.

### Factor analysis

**Figure 1:**
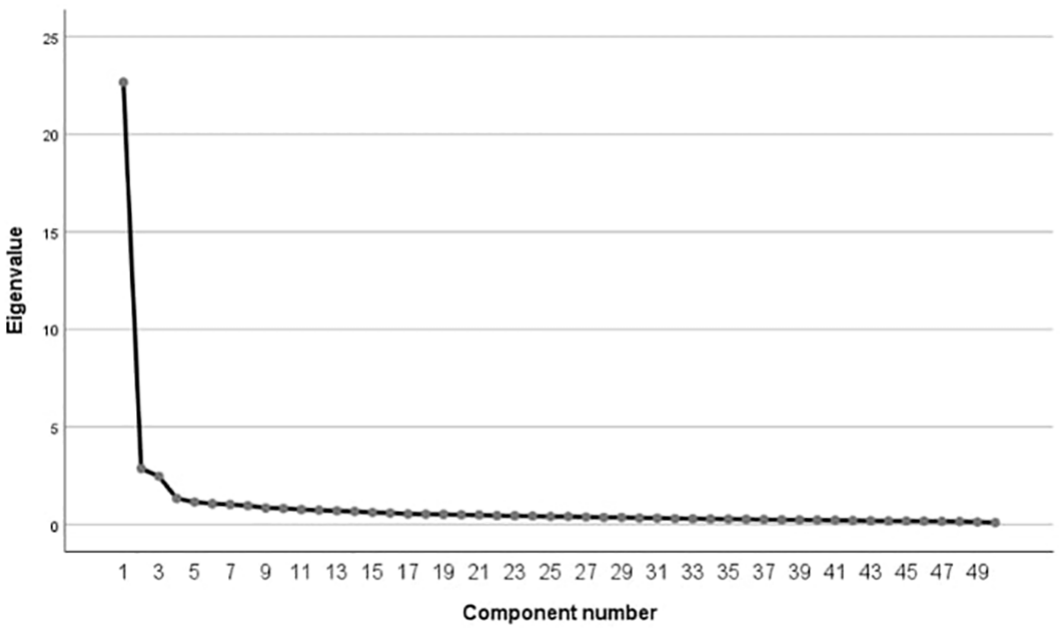
Scree plot for the exploratory factor analysis for the *Depersonalization Mechanism Scale*.

In order to determine psychometric values of the Depersonalization Mechanism Scale, principal component analysis with Oblimin rotation was performed. Sampling size was adequate—KMO = 0.96; Bartlett sphericity test [χ^2^(1,225) = 11,091.21; *p* < 0.001]. Seven factors had eigenvalue >1 (**Table 3**), but the scree plot showed three factors (**Figure 1**). Together, they explained 56.01% of variance.

**Table 3:** Component’s sum of squares after extraction and Oblimin rotation.

Component	All	% of variance	% cumulated	After rotation
1	22.66	45.33	45.33	20.53
2	2.88	5.75	51.08	16.74
3	2.47	4.93	56.01	2.64
4	1.34	2.68	58.69	
5	1.16	2.31	61.00	
6	1.07	2.14	63.13	
7	1.03	2.05	65.18	

Factors 1 and 2 were strongly, positively correlated (*r* = 0.61), and Factor 3 was not correlated either with Factor 1 (*r* = −0.04) or Factor 2 (*r* = 0.03). We decided to remove items from Factor 3 along with five other items loading two factors at the same time. The final version of the scale consists of 41 items, organized into two subscales: detachment and emotional numbness (factor loadings are presented in **Table 4**). Discriminatory power of the items is presented in **Table 5**.

**Table 4:** Factor loadings for the Depersonalization Mechanism Scale items.

	Factor	
	1	2
1.45. Familiar voices appear strange and peculiar	.88	
1.16. I have trouble fully feeling my own body	.87	
1.37. I have the experience of not being fully connected to my body	.86	
1.19. My surroundings seem far away and unclear, as if I were looking at it through a fog	.83	
1.10. I have the experience of being out of touch with my body	.82	
1.36. When I look in the mirror it is like I am looking at a stranger—I know it is me, but I do not feel emotionally connected to my reflection	.82	
1.08. My body feels strange	.79	
1.04. I have the experience of being outside of my body/watching myself from the distance	.77	
1.20. I catch myself being so invested in daydreaming that it interferes with my day-to-day life	.76	
1.29. I am so invested in fantasies that I feel like I am experiencing them for real	.75	
1.17. I have an impression as if my emotions and thoughts were not coherent with myself	.74	
1.38. When I experience something difficult, everything seems so “flat” and “faded”	.69	
1.11. In difficult times, I have the experience of being detached from my emotions	.68	
1.41. I have the experience of watching my life from the distance	.65	
1.05. Smells do not evoke neither pleasant nor unpleasant feelings	.64	
1.13. My thoughts seem to float uncontrollably	.63	
1.39. I realize that I have no feelings in situations when I would normally feel something	.63	
1.15. I have the experience of being “spaced out”	.61	
1.06. It seems as if my thoughts are outside of my control, as if they are not consistent with the rest of my experiences	.58	
1.24. I find myself treating others in an impersonal and almost automatic manner	.53	.34
1.02. I have trouble recalling happy memories, even though I know I have them	.52	
1.32. I realize how disconnected I am	.51	.35
1.07. I go through my day as if I am on autopilot and at times, I catch myself that I don’t remember what was happening	.50	
1.43. I have the experience of having a blank space in my head when I talk	.50	
1.44. When someone asks me a question, I feel as if I am answering automatically	.50	
1.42. The food has less distinct flavor	.48	
1.47. The fantasy world is a kind of escape from hard reality	.46	
1.26. I start distancing myself from everyone		.95
1.21. I do my business and I do not want to be bothered		.76
1.34. I feel emotionally distanced from others		.74
1.09. Other people seem unhelpful and ungrateful		.68
1.14. I try my best even though I am exhausted and yet nobody appreciates it		.67
1.28. It seems that people around me are particularly difficult		.65
1.18. I wish I had more control over my emotions		.64
1.49. When something particularly hard happens to me, I need a lot of time to gain balance again		.64
1.22. When things get hard, I become more and more cynical, relationships with others become less meaningful		.61
1.23. I only engage in what is necessary		.59
1.33. I have so little mental energy that I do the bare minimum when I interact with others		.58
1.50. I avoid contact with other people		.57
1.27. I feel emotionally detached from my surroundings	.32	.56
1.46. I start to distance myself and care very little about everything	.33	.54

**Table 5:** Discriminatory power for the Depersonalization Mechanism Scale items.

	Discriminatory power		
	Detachment	Emotional numbness	Depersonalization
1.45. Familiar voices appear strange and peculiar	.71		.50
1.16. I have trouble fully feeling my own body	.80		.62
1.37. I have the experience of not being fully connected to my body	.77		.67
1.19. My surroundings seem far away and unclear, as if I were looking at it through a fog	.82		.59
1.10. I have the experience of being out of touch with my body	.80		.65
1.36. When I look in the mirror it is like I am looking at a stranger—I know it is me, but I do not feel emotionally connected to my reflection	.74		.69
1.08. My body feels strange	.70		.57
1.04. I have the experience of being outside of my body/watching myself from the distance	.71		.62
1.20. I catch myself being so invested in daydreaming that it interferes with my day-to-day life	.76		.72
1.29. I am so invested in fantasies that I feel like I am experiencing them for real	.66		.48
1.17. I have an impression as if my emotions and thoughts were not coherent with myself	.82		.73
1.38. When I experience something difficult, everything seems so “flat” and “faded”	.79		.69
1.11. In difficult times, I have the experience of being detached from my emotions	.74		.75
1.41. I have the experience of watching my life from the distance.	.75		.75
1.05. Smells do not evoke neither pleasant nor unpleasant feelings.	.48		.65
1.13. My thoughts seem to float uncontrollably	.77		.76
1.39. I realize that I have no feelings in situations when I would normally feel something	.74		.73
1.15. I have the experience of being “spaced out”	.77		.78
1.06. It seems as if my thoughts are outside of my control, as if they are not consistent with the rest of my experiences.	.74		.77
1.24. I find myself treating others in an impersonal and almost automatic manner	.75		.71
1.02. I have trouble recalling happy memories, even though I know I have them	.59		.66
1.32. I realize how disconnected I am	.73		.68
1.07. I go through my day as if I am on autopilot and at times, I catch myself that I do not remember what was happening	.68		.73
1.43. I have the experience of having a blank space in my head when I talk	.68		.62
1.44. When someone asks me a question, I feel as if I am answering automatically	.67		.80
1.42. The food has less distinct flavor	.53		.78
1.47. The fantasy world is a kind of escape from hard reality	.57		.72
1.26. I start distancing myself from everyone		.68	.74
1.21. I do my business, and I do not want to be bothered		.72	.45
1.34. I feel emotionally distanced from others		.74	.77
1.09. Other people seem unhelpful and ungrateful		.67	.73
1.14. I try my best even though I am exhausted and yet nobody appreciates it		.70	.78
1.28. It seems that people around me are particularly difficult		.74	.74
1.18. I wish I had more control over my emotions		.61	.77
1.49. When something particularly hard happens to me, I need a lot of time to gain balance again		.66	.59
1.22. When things get hard, I become more and more cynical, relationships with others become less meaningful		.74	.76
1.23. I only engage in what is necessary		.53	.69
1.33. I have so little mental energy that I do the bare minimum when I interact with others		.73	.69
1.50. I avoid contact with other people		.70	.68
1.27. I feel emotionally detached from my surroundings		.73	.53
1.46. I start to distance myself and care very little about everything		.73	.58

Average variance extracted (AVE) and composite reliability (CR)/McDonald’s omega (37) scores were calculated for both subscales and the whole scale—are presented in **Table 1**. Average variance extracted in all of all three measures falls under the acceptable level of 0.50. However, according to Fornell and Larcker (38), AVE may be a more conservative estimate of the validity and “on the basis of p_n_ (composite reliability) alone, the researcher may conclude that the convergent validity of the construct is adequate, even though more than 50% of the variance is due to error” (p. 46). The composite reliability of all of the three constructs is above 0.90, so the internal reliability is deemed acceptable.

### Correlations

**Table 6** presents correlations between BERQ/CERQ and subscales of DMS (first and second column) and a whole scale (third column). Behavioral strategies positively correlated with depersonalization, except for actively approaching. Correlations ranged between 0.12 (seeking social support and depersonalization) and 0.70 (withdrawal and emotional numbness). The strongest correlations were found between all dimensions of depersonalization and withdrawal.

**Table 6:** Pearson correlations between depersonalization and cognitive and behavioral emotion regulation strategies.

		Detachment	Emotional numbness	Depersonalization
BERQ	Seeking distraction	.34**	.38**	.37**
	Withdrawal	.62**	.70**	.68**
	Actively approaching	.00	.01	0
	Seeking social support	.15*	.03	.12*
	Ignoring	.50**	.41**	.49**
CERQ	Self-blame	.46**	.53**	.51**
	Acceptance	.37**	.45**	.42**
	Rumination	.48**	.59**	.54**
	Positive refocusing	.26**	.17*	.24**
	Refocus on planning	.10	.23**	.15*
	Positive reappraisal	.15*	.13*	.15*
	Putting into perspective	.20**	.23**	.22**
	Catastrophizing	.52**	.56**	.56**
	Blaming others	.51**	.46**	.52**

*p< 0.05, **p< 0.001; BERQ, Behavioral Emotion Regulation Questionnaire; CERQ, Cognitive Emotion Regulation Questionnaire. Statistically significant results are marked in bold.

All relationships between depersonalization and cognitive strategies were significant, except the one between refocus on planning and detachment. Correlations ranged between 0.13 (positive reappraisal and emotional numbness) and 0.59 (rumination and emotional numbness). The strongest correlations were found between all dimensions of depersonalization and two subscales of CERQ—rumination and catastrophizing.

**Table 7** presents correlations between depersonalization and occupational depression, depressive symptoms, and trait anxiety. Correlations ranged between 0.47 (detachment and anxiety**—**trait) and 0.69 (depressive symptoms and depersonalization). All the correlations were significant; the strongest relationship was found between all aspects of depersonalization and depressive symptoms.

**Table 7:** Pearson correlations between depersonalization and depression (measured by ODI and PHQ) and anxiety trait.

	Detachment	Emotional numbness	Depersonalization
Occupational depression (ODI)	.57**	.50**	.58**
Depression (PHQ)	.66**	.65**	.69**
Anxiety trait (SL-C)	.47**	.56**	.53**

**p< 0.001; ODI, Occupational Depression Inventory; PHQ, Patient Health Questionnaire; SL-C, Trait Anxiety Scale. Statistically significant results are marked in bold.

### Gender differences in depersonalization and emotion regulation strategies

Student’s *t*-test was performed in order to determine whether men and women differ in severity of depersonalization and emotion regulation strategies. Differences in the aspect of emotional numbness (*t* = 3.12; *p* = 0.002) and depersonalization (*t* = 2.04; *p* = 0.043) were found to be significant, with women being more prone than men to both. However, the effect size was small (*d* = 0.36 and *d* = 0.24, respectively). There were no differences in the aspect of detachment.

Behavioral and cognitive emotion regulation strategies were also considered. In our sample, women were more likely than men to use the following behavioral strategies: withdrawal (*t* = 2.37; *p* = 0.019), actively approaching (*t* = 2.48; *p* = 0.014), and seeking social support (*t* = 2.72; *p* = 0.007); other differences were insignificant. However, the effect size was small (*d* = 0.27, *d* = 0.29, *d* = 0.31, respectively). Regarding cognitive strategies, in our sample, women were more likely to use the following strategies: self-blame (*t* = 2.36; *p* = 0.019), acceptance (*t* = 2.65; *p* = 0.009), rumination (*t* = 4.73; *p*< 0.001), and refocus on planning (*t* = 2.20; *p* = 0.029 and catastrophizing (*t* = 3.33; *p* = 0.001); other differences were insignificant. Effect sizes were small (*d* = 0.27, *d* = 0.31, *d* = 0.25, *d* = 0.38, respectively), except for rumination, where the effect was medium (*d* = 0.50).

### Multiple regression

Multiple regression analyses were conducted to test if behavioral and cognitive emotion regulation strategies significantly predicted depersonalization. The results of the regression indicated that behavioral emotion regulation strategies explained 52% of the variance in depersonalization [*R^2^* ^Adjusted^ = .52, *F*(5,294) = 65.75, *p* < 0.001]. It was found that withdrawal (*β* = .38, *p* < 0.001), ignoring (*β* = .19, *p* < 0.001), and seeking distraction (*β* = .10, *p* = 0.038) significantly predicted depersonalization (**Table 8**).

**Table 8:** Regression coefficients of behavioral emotion regulation strategies on depersonalization.

Variables	*B*	*SE*	*t*	*p*	95% CI
Constant	−.094	.135	−0.699	0.485	[−.359,.171]
Seeking distraction	.101	.048	2.089	0.038	[.006,.196]
Withdrawal	.381	.033	11.584	<0.001	[.316,.446]
Actively approaching	−.072	.039	−1.869	0.063	[−.148,.004]
Seeking social support	.067	.039	1.728	0.085	[−.009,.143]
Ignoring	.190	.039	4.865	<0.001	[.113,.267]
Seeking distraction	−.094	.135	−0.699	0.485	[−.359,.171]

CI, confidence interval.

Further analysis revealed that cognitive emotion regulation strategies are significant predictors of depersonalization and explained 42% of the variance [*R^2^* ^Adjusted^ = .42, *F*(9,290) = 25.45, *p* < 0.001]. It was found that self-blame (*β* = .21, *p* < 0.001), blaming others (*β* = .25, *p* < 0.001), rumination (*β* = .23, *p* = 0.002), and refocus on planning (*β* = −.15, *p* = 0.009) were significant predictors of depersonalization (**Table 9**).

**Table 9:** Regression coefficients of cognitive emotion regulation strategies on depersonalization.

Variables	*B*	*SE*	*t*	*p*	95% CI
Constant	−.304	.154	−1.980	0.049	[−.607, −.002]
Self-blame	.209	.062	3.373	<0.001	[.087,.330]
Acceptance	.061	.065	0.939	0.349	[−.067,.189]
Rumination	.226	.073	3.096	0.002	[.082,.370]
Positive refocusing	.013	.062	0.210	0.834	[−.109,.135]
Refocus on planning	−.145	.055	−2.626	0.009	[−.254, −.036]
Positive reappraisal	.014	.062	0.218	0.828	[−.109,.136]
Putting into perspective	.000	.063	−0.006	0.995	[−.124,.123]
Catastrophizing	.107	.066	1.610	0.108	[−.024,.237]
Blaming others	.250	.056	4.429	<0.001	[.139,.361]

CI, confidence interval.

Subsequently, the two factors of depersonalization, i.e., detachment and emotional numbness, were tested to evaluate the extent to which depersonalization could predict symptoms of depression. The results of the regression analysis indicated that symptoms of depersonalization explained 48% of the variance [*R^2^* ^Adjusted^ = .48, *F*(2,297) = 137.37, *p* < 0.001]. It was found that both detachment (*β* = .35, *p* < 0.001) and emotional numbness (*β* = .33, *p* < 0.001) significantly predicted depression (**Table 10**).

**Table 10:** Regression coefficients of symptoms of depersonalization on depression (PHQ).

Variables	*B*	*SE*	*t*	*p*	95% CI
Constant	−.146	.077	−1.903	0.058	[−.297, −.005]
Detachment	.349	.061	5.692	<0.001	[.229,.470]
Emotional numbness	.331	.062	5.347	<0.001	[.209,.453]

CI, confidence interval; PHQ, Patient Health Questionnaire.

A significant regression was also found in the context of occupational depression [*R^2^* ^Adjusted^ = .33, *F*(2,223) = 56.34, *p* < 0.001], indicating that depersonalization explained 33% of the variance (**Table 11**). In this analysis, only detachment was found as a significant predictor for occupational depression (*β* = .44, *p* < 0.001).

**Table 11:** Regression coefficients of symptoms of depersonalization on occupational depression (ODI).

Variables	*B*	*SE*	*t*	*p*	95% CI
Constant	−.027	.097	−0.275	0.783	[−.218,.165]
Detachment	.439	.081	5.422	<0.001	[.280,.599]
Emotional numbness	.116	.080	1.437	0.152	[−.043,.274]

CI, confidence interval; ODI, Occupational Depression Inventory.

## Discussion

The aim of this paper was to introduce our understanding of depersonalization mechanism and its relationship with selected emotion regulation depression and anxiety. Moreover, our purpose was to investigate whether depersonalization acts as a predictive factor for depressive symptoms.

This work proposes a new measure that offers a deeper understanding of this complex phenomenon. The constructed method, the Depersonalization Mechanism Scale (DMS), comprises two subscales, namely, detachment and emotional numbness, both of high reliability (*α* = 0.97 and *α* = 0.94, respectively). The whole scale was also found to be highly reliable (*α* = 0.97) and applicable to adults in non-clinical population.

We aimed to shed light on depersonalization outside of strictly clinical context and normalize it as one of the possible mechanisms of emotional regulation. We propose that depersonalization mechanism is a non-voluntary, complex reaction that entails specific cognitive and behavioral strategies. The maladaptive function of depersonalization may be suggested by stronger correlation with less functional cognitive strategies, namely, self-blame, blaming others, rumination, and catastrophizing, and behavioral ones, namely, withdrawal and ignoring. However, due to significant correlations with more adaptive strategies (acceptance, positive refocusing, putting into perspective, positive reappraisal, seeking distraction and seeking social support), we may conclude that there are some positive aspects of depersonalization indicating adaptive characteristics of this mechanism. Depersonalization may provide short-term relief, especially important when we do not have the ability to change the situation that we must endure. The most important aspect of determining the adaptability of a given mechanism is determined by flexibility and adequacy of use.

It is worth noting that depersonalization cannot be reduced to avoidance only, which is confirmed by correlations with various emotion regulation strategies. This leads us to the conclusion that depersonalization is a complex mechanism of emotional regulation. Additionally, causes and motivation distinguish depersonalization from avoidance. Avoidant personality disorder is driven by feelings of inadequacy and intense fear of rejection (16), avoidance-based emotion regulation strategies are used to escape from unpleasant experiences (13), while depersonalization could be understood as a kind of “energy conservation” mechanism. We believe that adaptivity of such strategies is dependable on flexibility of use, the amount of control over it, the severity of consequences, among others.

Such perspective on depersonalization may lead to normalization of this specific response and help to better understand seemingly inadequate behaviors and attitudes. Furthermore, it may influence support and psychoeducation in the therapeutic interventions in patients who experience acute or chronic stress. The need to raise awareness among practitioners and the general population is particularly important in the light of the apparent rise in depersonalization symptoms (39, 40). Alterations in the sense of self with all of its consequences may constrain people’s ability to be more present and engaged in their lives. One of the possible results could be gradual drainage of social support network, essential for maintaining a sense of self (40).

Multiple regression analyses showed that behavioral and cognitive strategies play an important role in explaining depersonalization mechanism. The behavioral strategies explained a greater percentage of variance in depersonalization than cognitive ones. Among behavioral strategies, withdrawal, ignoring, and seeking distraction were significant predictors of depersonalization—the greater the level of these strategies, the stronger the symptoms of depersonalization. Cognitive strategies in regulating emotions revealed the similar pattern in relation to maladaptive strategies, namely, self-blame, blaming others, and rumination; the only one adaptive strategy that predicted the depersonalization was refocus on planning, which was negatively associated with depersonalization. It seems that planning may be more available in depersonalization than other adaptive cognitive strategies, like positive refocusing or positive reappraisal. This analysis helps to indicate specific emotion regulation strategies that may have a particular influence on strengthening depersonalization mechanism (i.e., withdrawal, ignoring, seeking distraction, self-blame, blaming others, and rumination), increasing the tendency to react in line with depersonalization characteristics and one cognitive strategy (i.e., refocus on planning) that may be crucial in limiting the tendency to depersonalization.

Another inspiring outcome of our analysis refers to the symptoms of depersonalization as predictors of depression. Gathered data indicate that, in a general context, both detachment and emotional numbness increase the symptoms of depression. Considering work-related context, it seems that detachment may play a crucial role in the development of occupational depression. Interestingly, “detachment” has stronger cognitive and behavioral connotations and is more prone to change than the “emotional numbness,” which is related stronger to emotions and the states caused by them. Expanding research on depersonalization in non-clinical groups could be beneficial for our understanding of this phenomenon. Combining this knowledge with increased awareness of non-clinical depersonalization could help in the development of preventive actions against depression and provide better professional support for those experiencing acute or prolonged stressful life situations.

There are several limitations to our study. We focused primarily on the description and characteristics of depersonalization in non-clinical population. Further studies should incorporate clinical samples analyzing the problem of depersonalization mechanism in different disorders. It is very important to identify individual dispositions (e.g., temperament, personality, sensory processing sensitivity) and contextual factors (e.g., family ties, traumas, social support) as possible predictors of depersonalization mechanism. In this study, basic correlations with anxiety and depression are reported; however, cause-and-effect relationships need to be studied to describe possible functions and consequences. Finally, further research on neurophysiological correlates of depersonalization can bring insight into the fundamental brain mechanisms. It would be especially valuable to use an experimental model to study different aspects of the depersonalization mechanism, like information processing in different conditions and stimuli characteristics.

The results of this study may be used in intervening programs, which could focus on developing skills in reducing strategies such as withdrawal, ignoring, distraction, blaming oneself or others, rumination on the one hand, and intensifying planning strategies on the other. It seems that precise selection of these strategies may allow for more accurate therapeutic interventions to reduce the tendency to depersonalization. It is particularly important in the light of the strong association between depersonalization and depression symptoms, both in non-professional and occupational contexts. Understanding the mechanisms of depersonalization may benefit in weakening this tendency with regard to strength and duration of being detached and emotionally numbed. The consciousness of potential depressive consequences seems to be a sufficient reason to deepen knowledge about the mechanisms of depersonalization.

## Data Availability

The raw data supporting the conclusions of this article will be made available by the authors, without undue reservation.
