# Supplementary material for: When you avoid your feelings, you may feel even worse: how depersonalization puts you at risk of depression
Source: Front Psychiatry. 2024 Oct 17;15:1481439. doi: 10.3389/fpsyt.2024.1481439 (PMC11528536; doi:10.3389/fpsyt.2024.1481439)
Supplement: Supplementary file 1 [file Table1.docx]

***Supplementary Materials***

**DEPERSONALIZATION MECHANISM SCALE**

Think about a difficult time in your life – it may concern day-to-day situations, relationships or a specific context (for example your job) and recall how you were feeling at that time. Read those sentences and assess to what extent those states and reactions* are typical for you when you are overwhelmed.

**NOT caused by psychoactive substances or medication*

0 – never

1 – rarely

2 – sometimes

3 – often

4 – very often

1. I have trouble recalling happy memories, even though I know I have them.

2. I have the experience of being outside of my body/ watching myself from the distance.

3. Smells do not evoke neither pleasant nor unpleasant feelings.

4. It seems as if my thoughts are outside of my control, as if they are not consistent with the rest of my experiences.

5. I go through my day as if I am on autopilot and at times, I catch myself that I don't remember what was happening.

6. My body feels strange.

7. Other people seem unhelpful and ungrateful.

8. I have the experience of being out of touch with my body.

9. In difficult times, I have the experience of being detached from my emotions.

10. My thoughts seem to float uncontrollably.

11. I try my best even though I am exhausted and yet nobody appreciates it.

12. I have the experience of being “spaced out”.

13. I have trouble fully feeling my own body.

14. I have an impression as if my emotions and thoughts were not coherent with myself.

15. I wish I had more control over my emotions.

16. My surroundings seem far away and unclear, as if I were looking at it through a fog.

17. I catch myself being so invested in daydreaming that it interferes with my day-to-day life.

18. I do my business and I don’t want to be bothered.

19. When things get hard, I become more and more cynical, relationships with others become less meaningful.

20. I only engage in what is necessary.

21. I find myself treating others in an impersonal and almost automatic manner.

22. I start distancing myself from everyone.

23. I feel emotionally detached from my surroundings.

24. It seems that people around me are particularly difficult.

25. I am so invested in fantasies that I feel like I am experiencing them for real.

26. I realize how disconnected I am.

27. I have so little mental energy that I do the bare minimum when I interact with others.

28. I feel emotionally distanced from others.

29. When I look in the mirror it is like I am looking at a stranger – I know it is me, but I don’t feel emotionally connected to my reflection.

30. I have the experience of not being fully connected to my body.

31. When I experience something difficult, everything seems so “flat” and “faded”.

32. I realize that I have no feelings in situations when I would normally feel something.

33. I have the experience of watching my life from the distance.

34. The food has less distinct flavour.

35. I have the experience of having a blank space in my head when I talk.

36. When someone asks me a question, I feel as if I am answering automatically.

37. Familiar voices appear strange and peculiar.

38. I start to distance myself and care very little about everything.

39. The fantasy world is a kind of escape from hard reality.

40. When something particularly hard happens to me, I need a lot of time to gain balance again.

41. I avoid contact with other people.

*Detachment*: 1, 2, 3, 4, 5, 6, 8, 9, 10, 12, 13, 14, 16, 17, 21, 25, 26, 29, 30, 31, 32, 33, 34, 35, 36, 37, 39

*Emotional numbness:* 7, 11, 15, 18, 19, 20, 22, 23, 24, 27, 28, 38, 40, 41
